# Non-typhoidal *Salmonella* in the Pig Production Chain: A Comprehensive Analysis of Its Impact on Human Health

**DOI:** 10.3390/pathogens8010019

**Published:** 2019-01-29

**Authors:** Joana Campos, Joana Mourão, Luísa Peixe, Patrícia Antunes

**Affiliations:** 1 UCIBIO@REQUIMTE, Laboratório de Microbiologia, Faculdade de Farmácia, Universidade do Porto, Portugal; Rua de Jorge Viterbo Ferreira nº 228, 4050-313 Porto, Portugal; joanaskila@hotmail.com (J.C.); joanavm1@gmail.com (J.M.); lpeixe@ff.up.pt (L.P.); 2 Faculdade de Ciências da Nutrição e Alimentação, Universidade do Porto, Portugal; Rua Dr. Roberto Frias, 4200 Porto, Portugal

**Keywords:** *Salmonella*, pig production, pork meat, foodborne transmission, antimicrobial resistance, clones, *S.* Typhimurium, *S.*1,4,[5],12:i:-, *S.* Derby, *S.* Rissen

## Abstract

Salmonellosis remains one of the most frequent foodborne zoonosis, constituting a worldwide major public health concern. The most frequent sources of human infections are food products of animal origin, being pork meat one of the most relevant. Currently, particular pig food production well-adapted and persistent *Salmonella enterica* serotypes (e.g., *Salmonella* Typhimurium, *Salmonella* 1,4,[5],12:i:-, *Salmonella* Derby and *Salmonella* Rissen) are frequently reported associated with human infections in diverse industrialized countries. The dissemination of those clinically-relevant *Salmonella* serotypes/clones has been related to the intensification of pig production chain and to an increase in the international trade of pigs and pork meat. Those changes that occurred over the years along the food chain may act as food chain drivers leading to new problems and challenges, compromising the successful control of *Salmonella*. Among those, the emergence of antibiotic resistance in non-typhoidal *Salmonella* associated with antimicrobials use in the pig production chain is of special concern for public health. The transmission of pig-related multidrug-resistant *Salmonella* serotypes, clones and/or genetic elements carrying clinically-relevant antibiotic resistance genes, frequently associated with metal tolerance genes, from pigs and pork meat to humans, has been reported and highlights the contribution of different drivers to the antibiotic resistance burden. Gathered data strengthen the need for global mandatory interventions and strategies for effective *Salmonella* control and surveillance across the pig production chain. The purpose of this review was to provide an overview of the role of pig and pork meat in human salmonellosis at a global scale, highlighting the main factors contributing to the persistence and dissemination of clinically-relevant pig-related *Salmonella* serotypes and clones.

## 1. Introduction

*Salmonella enterica* infections are a worldwide major public health concern, specifically human salmonellosis caused by non-typhoidal *Salmonella* (NTS) [1,2]. Salmonellosis is typically characterized by a self-limiting gastroenteritis syndrome, with diarrhea as its the main symptom; however, fever, vomiting and abdominal pain can also occur [1,2]. Despite being uncommon, more severe invasive *Salmonella* infections, as bacteraemia and/or other extra-intestinal infections, can occur and affect particular high-risk groups (infants, young children, older people or immunocompromised patients) [1,2]. In these cases, the use of antimicrobial agents is required, being of concern the emergence of *Salmonella* resistant to antibiotics, especially those considered by the World Health Organization (WHO) as “highest priority critically important antimicrobials” such as fluoroquinolones and extended-spectrum cephalosporins that can compromise the effective treatment of infections [1,2,3].

In industrialized countries, the main reservoir of NTS is the gastrointestinal tract of warm-blooded animals, in particular food-producing animals, which lead to foodstuffs contamination [1,2]. Therefore, the ingestion of contaminated food, particularly foods of animal origin, is recognized as the most relevant source of transmission of NTS to humans, with a high global impact in human health [2]. NTS causes an estimated 93.8 million cases of human illnesses and 155.000 deaths each year worldwide [1,2]. In the United States of America (USA), the 2015 report from the Centers for Disease Control and Prevention (CDC) showed that *Salmonella* was the second foodborne pathogen responsible for outbreaks (34%), being the first associated with outbreak illnesses (39%), hospitalizations (64%) and deaths (60%) [4]. Additionally, the European Food Safety Authority (EFSA) reported that salmonellosis has been the second most common zoonosis (91,662 confirmed salmonellosis cases in 2017) and the most frequent cause of foodborne outbreaks (24,4% of all cases in 2017) in the European Union (EU), in spite of a decreasing number of cases since 2008, with a stabilizing trend between the years 2013–2017 [5]. Salmonellosis has been mostly associated with the consumption of poultry products, including eggs and egg products, at a global level [2,5,6,7,8,9,10]. However, pork meat has been considered one of the major food products of animal origin responsible for *Salmonella* transmission to humans in diverse countries, including industrialized ones [2,11,12,13]. In the EU, pork meat has been a common source of human salmonellosis cases (varying from 2% to 13%), after eggs and egg products [5,6,7,8]. Additionally, in the USA (2015 data), pork meat was the second source attributed to *Salmonella* outbreaks (5%) and the meat product mostly associated with the largest number of illnesses (16%), hospitalizations (2%) and deaths (11%) [4].

Although different serotypes have been associated with salmonellosis, the major ones responsible for human infections in diverse industrialized countries include *Salmonella* Enteritidis, *Salmonella* Typhimurium and its monophasic variant—*Salmonella* 1,4,[5],12:i:- [4,5,14,15,16,17]. At a global level, *S.* Enteritidis is commonly associated with poultry and products thereof, being considered a poultry-related serotype [2,5,9]. In contrast, *S.* Typhimurium has a wider host range, including pigs [1,2,5,6]. Nevertheless, in the last decades, a changing trend in *Salmonella* serotypes associated with foodborne salmonellosis has been observed, with the worldwide expansion of previously less common serotypes (e.g., *S.* 1,4,[5],12:i:-, *Salmonella* Derby and *Salmonella* Rissen). Those are currently well-known serotypes associated with pig production chain and frequently multidrug-resistant [5,6,11,16,18,19,20,21,22,23,24,25,26,27,28].

In the EU, the successful implementation of mandatory *Salmonella* control programmes along poultry/egg production chain was responsible for the reduction of the prevalence of *Salmonella* serotypes considered relevant for public health, particularly *S.* Enteritidis [5,6,29]. In contrast to poultry production, where *Salmonella* control programmes are harmonized, for pig production each EU member state applies a specific national monitoring programme [5,6,13]. In addition, the intensive food production/farming and increased globalization of food supply (live animals and foodstuffs), with pork meat being one of the most consumed and traded meat products (pork exports increased in value by 18.2% from 2015 to 2016) [30,31], may trigger new problems regarding salmonellosis control. This review will provide evidence of the relevant role of pig production and pork meat in salmonellosis on a global scale.

## 2. Non-Typhoidal *Salmonella* in Pigs and Pig Production Chain

The colonization of pig populations with NTS is frequent and normally results in asymptomatic healthy carriers. This pig colonization can occur throughout all stages of pig production chain by horizontal (through external agents in the environment, e.g., rodents, birds, people, trucks, pets, other foodstuffs) and vertical transmission (e.g., from sow to piglet and from pig to pig at herd to slaughter). Additionally, a designated circular transmission (combination of vertical and horizontal transmission), which is a permanent cycle of contamination on a farm (e.g., environmental contamination through pig shedding and pig contamination through farm environment) can also determine pig colonization [11,13,32,33,34]. The presence of *Salmonella* in those healthy pig carriers (e.g., tonsils, gut and gut-associated lymphoid tissue) is suggested to be the main risk factor for the spread and transmission of these bacteria across pig production chain to humans: in pre-harvest (holding period of pig on the farm), in the harvest stage (during slaughter and further processing of meat carcasses) and in the post-harvest stage (during final preparation of pork meat and products thereof) [11,13,32,33,35,36].

In fact, diverse studies aiming to detect *Salmonella* in the pig production chain, including pigs and pork meat, have been performed in high or low-income countries with diverse results. In the EU, data from the last EFSA reports revealed *Salmonella*-positive samples in fresh pork meat (2.4%-2016 and 1.6%-2017) and products thereof (1.9%-2016), with an overall *Salmonella* prevalence in pigs of 6.7% at the herd (ranging from 0%-63% between the different member states, 2016) and of 3.5% (2016) and 12.7% (2017) at slaughter [5,6]. A noteworthy, high prevalence of *Salmonella* in fecal samples (30.5%), rectal swabs (24%) and carcass swabs (9.6%) of slaughter pigs was reported by a United Kingdom (UK) study [37]. In contrast, other EU countries (Finland, Sweden and Norway), with special guarantees concerning *Salmonella* on pig carcasses (according to Regulation (EC) No 853/2004) reported a lower incidence of *Salmonella* in pig carcasses samples (0.02%) [6,38]. With the objective to establish the main targets for *Salmonella* reduction in breeding herds of pigs (in line with the Regulation (EC) No 2160/2003) [29], a baseline survey was performed in EU (2008) due to the lack of information about *Salmonella* control in holdings of breeding pig [32]. In this survey, high levels of *Salmonella*-positive holdings with breeding pigs (31.8%) and breeding holdings (28.7%) were observed. These data have shown that breeding pigs may be a relevant source of *Salmonella* dissemination along the pig production chain (e.g., to slaughter pigs through trade and movement of live animals and contamination of holding, transport, lairage and slaughter facilities), leading to pork meat contamination and consequently to human infections [32]. In fact, high levels of *Salmonella* contamination occurred in slaughterhouses by diverse routes (e.g., pork meat carcasses cross-contamination, slaughter environment and equipment, meat handlers), as reported in diverse studies [11,13,39]. Another EU survey based on the analysis of quantitative microbiological risk assessment of *Salmonella* in slaughter and breeder pigs showed that an 80–90% reduction of *Salmonella* prevalence in lymph nodes should result in a comparable reduction in the number of human cases attributable to pork meat products [40]. Consequently, several interventions have been proposed in order to prevent or reduce *Salmonella* contamination, persistence and dissemination across pig production (at farm and slaughterhouse). These included the use of uncontaminated feed, isolation of newly purchased animals before introducing them into herd, regular veterinary checks, vaccination, prevention of environmental contamination at the farm, transport, lairage and slaughter, implementation of high standards of hygiene (cleaning and disinfection) and the promising alternative approach bacteriophage use [11,13,32,34,40,41]. Starting 2006, in all EU member states, the report of data regarding *Salmonella* monitoring and surveillance became mandatory under the Commission Regulation (EC) No 2073/2005 on microbiological criteria in foodstuffs, including for *Salmonella* in pig carcasses at dressing and before chilling stage. Furthermore, competent authorities must verify the correct implementation of the process hygiene criteria for *Salmonella* on pig carcasses by food business operators and, if it is not complied, business operators might implement corrective actions (e.g., improvements of hygiene slaughter, biosecurity measures in the farms and revision of process controls) with the specific instructions of authorities [42]. In 2014, legislation was revised, with Regulation (EC) No 217/2014 proposing the reduction of *Salmonella* acceptable number in pig carcasses, from “c = 5 out of n = 50”—10% to “c = 3 out of n = 50”—6% (n-number of units comprising the sample; c- detection number of samples with *Salmonella*), in order to strengthen the process hygiene criterion [43]. 

In the USA, another high-income country, data from 2015 showed a remarkable higher prevalence of *Salmonella* in pig fecal samples (50%-sows and 35%-market swine) than in other animal samples (25%-chickens, 9%-turkeys, 22%-dairy and 8%-beef) [44]. Moreover, in developing countries (some with an expansion of food-animal industry), *Salmonella* was detected at high levels in pig samples (animal, carcasses and meat), ranging from 17–39% in South America [45,46], to 14–40% in Africa [47,48] and 29–100% in Asia [49,50]. Those high levels possibly reflect the different pig production practices and the absence of control measures. Overall, these data point out the need of implementing effective global measures for *Salmonella* control, highlighting the need for its detection at all stages of pig production chain, including in primary production [13,32,33]. This is particularly urgent in developing countries, which currently seem to present a severely underserved monitoring surveillance program [51].

## 3. Major Pig-Related *Salmonella* Serotypes Associated with Human Infections

In recent years, *Salmonella* transmission from pigs to humans through the pork food chain has been evidenced, namely through the study of serotypes prevalence in different matrices as well as food-borne outbreaks associated with consumption of pork products. 

**Figure 1:**
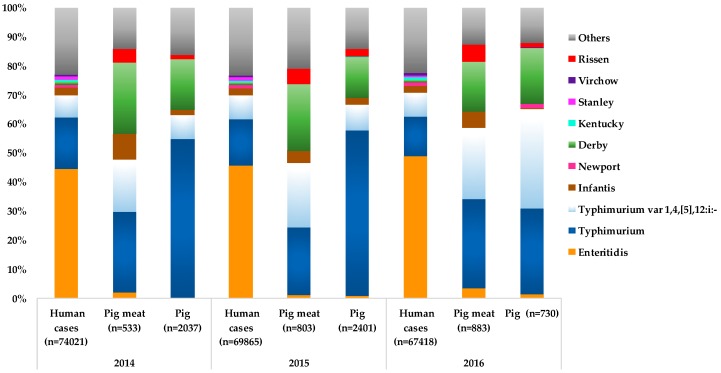
Distribution of the major serotypes of non-typhoidal *Salmonella* associated with human cases (salmonellosis), pig and pig meat in EU, 2014 to 2016 [6,7,8]. *S.* Rissen was included for being one of the five most frequent *Salmonella* serotypes recovered from pig meat and pig animal in EU, 2014 to 2016 [6,7,8]. The percentages were calculated based on the total number of serotyped isolates (represented by the numbers in brackets) per human salmonellosis cases, pig meat or pig animal.

Worldwide data concerning the prevalence of *Salmonella* serotypes in humans, pigs and products thereof have contributed to establishing their epidemiological correlation, with particular serotypes overlapping between humans, pig and pork meat [6,7,8,14,16,17,52,53,54]. For instance, in EU, an association between *Salmonella* serotypes causing human infections and those occurring in pig and pork meat was observed (Figure 1), reinforcing the major role of pork meat in the transmission of *Salmonella* to humans. The most frequent serotypes in pig and pork meat have been *S.* Typhimurium (pig: 54.7%-2014, 56.9%-2015, 29.5%-2016 and 20.6%-2017; pork meat: 27.8%-2014, 23%-2015, 30.7%-2016 and 27%-2017), *S.* 1,4,[5],12:i:- (pig: 8.4%-2014, 8.6%-2015, 34.1%-2016 and 37.4%-2017; pork meat: 18%-2014, 22.3%-2015, 24.3%-2016 and 22%-2017), and *S.* Derby (pigs: 17.5%-2014, 13.7%-2015 and 19.2%-2016; pork meat: 24.4%-2014, 22.9%-2015 and 17%-2016) [5,6,7,8]. These three serotypes are also among the major ones associated with human salmonellosis (second-, third- and fifth-ranked, respectively, in 2014-2016) [6,7,8]. From the 2008 EU baseline survey, *S.* Derby was the most frequent serotype found in both breeding (29.6%) and production holdings (28.5%) and *S.* Typhimurium was the second most detected (breeding holdings-25.4% and production holdings-20.1%) [32]. It is also of note the emergence of *S.* Rissen in pig sources (pigs: 1.5%-2014, 2.8%-2015 and 1.2%-2016; pork meat: 4.9%-2014, 5.1%-2015 and 5.9%-2016) in the EU (the fifth most common serotype since 2014 in pig sources) in spite of its low association with human infections (Figure 1) [6,7,8]. The EU baseline survey reported a high incidence of *S.* Rissen in breeding and production holdings, particularly in Portugal (40% and 22.4%, respectively) and Spain (25% and 29.7%, respectively), being the first or second most frequently reported serotype in those settings [32,33]. In fact, in Portugal, *S.* Rissen was the fourth most frequently serotype detected in human clinical isolates between 2002 and 2016 [55,56]. Although *S.* Enteritidis (number one in human infections) is typically associated with eggs and poultry meat, it is important to point out that in the last years, in the EU this serotype was also common in both pig and pork meat samples (varying from 1% and 3.5%) [5,6,7,8]. Moreover, *Salmonella* Infantis, another typically poultry-related serotype causing human infections (top 4), was detected in pigs and particularly in pork meat (varying from 3.9% to 8.8%) [5,6,7,8]. Therewithal, both *S.* Enteritidis and *S.* Infantis serotypes have been recovered from pigs/pork and products thereof in other non-EU regions and associated with human salmonellosis [57,58,59,60,61]. 

## 4. Dissemination of Pig-Associated *Salmonella* Serotypes and Clones

Besides worldwide data concerning *Salmonella* serotypes prevalence in humans and pig sources, the contribution of pork meat for human salmonellosis has been also evidenced throughout the spread of certain pig-associated strains and clones. Several examples of outbreaks associated with pig-related *Salmonella* serotypes have been described involving diverse countries (Table 1). For instance, *S.* 1,4,[5],12:i:- strains causing human infections, including some particular major clones, were identified in diverse European countries and associated with the consumption of different pork products (Table 1). Moreover, since 2015, several notifications were reported by the Rapid Alert System for Food and Feed (RASFF) due to the presence of *Salmonella* in pork products from several European countries, including alerts of suspected multi-country foodborne outbreaks (e.g., *S.* Typhimurium ST19 in Denmark associated with chilled sliced salami from Spain) (Table 1). This scenario alerts for the relevant role of pig/pork meat international trade on the dissemination of clinically-relevant pig-related *Salmonella* serotypes/clones, highlighting the need for global effective surveillance and detection programmes at all stages of pig production [11,13].

Regarding *S.* Typhimurium, diverse examples of pork products-related outbreaks have been reported in the last years, highlighting the importance of this serotype in the pig production chain (Table 1). Moreover, during the last decade, *S.* Typhimurium has been associated with clinically-relevant multi-drug resistant (MDR) clones, being of note the globally disseminated *S.* Typhimurium DT104 phagetype clone/sequence type (ST by MLST) 19, already reported associated with pig production [25,62,63,64,65,66]. In the same way, MDR *S.* Typhimurium OXA-30-producing/ST19 associated with swine production samples was also reported in Portugal and other European countries [63,67,68,69]. More recently, *S.* Typhimurium European clone/ST34 with a MDR-profile (ASSuT; Ampicillin-A, Streptomycin-S, sulphonamides-Su and Tetracycline-T) and Pulsed-Field Gel Eletrophoresis (PFGE)-types similar to *S.* 1,4,[5],12:i:- European clone, were reported in Europe, particularly among piggeries, abattoirs, pork meat as well as human infections [24,62,65,70]. 

In the EU, *S.* 1,4,[5],12:i:- is considered an emergent serotype that causes human infections [71,72,73], with a remarkable increase in the incidence in pig and pork, surpassing even in the last years *S.* Typhimurium [5,6,7,8]. In fact, recent European surveys have demonstrated a high prevalence of *S.* 1,4,[5],12:i:- in pigs, carcass and environmental samples (14% to 43%) [19,74]. Furthermore, several studies have already demonstrated the same clonal relatedness between *S.* 1,4,[5],12:i:- isolates from human clinical and pigs and/or products thereof [19,24,62,72,75], which evidence that pigs are the main animal reservoir of this emerging serotype in European countries [6,24,62,72]. Interestingly, a remarkable increase of this serotype in human clinical cases was observed in Portugal, from third- (4.5%, between 2000 and 2012) [55] to first-ranked serotype (36.6%, in 2014 to 2016) [56], surpassing *S.* Enteritidis and *S.* Typhimurium. In addition to *S.* Typhimurium, *S.* 1,4,[5],12:i:- was, in recent years, the other major serotype responsible for large outbreaks associated with diverse pork products (Table 1). Moreover, two predominant MDR clones of *S.* 1,4,[5],12:i:-, the European clone/ST34 and the Spanish clone/ST19, have been recognized as responsible for most human infections through pork products. The European clone, frequently belonging to DT120 and DT193 phage types has been circulating in several regions of Europe [70,71,76,77] and more recently in America [78,79], Asia [18] and even Australia [28]. Meanwhile, the Spanish clone, with most of the isolates belonging to DT104/U302 phage types, was originally identified in Spain and further reported since 2002 in the Iberian Peninsula [24,62,80,81,82]. The maintenance and dissemination of these MDR clones in Europe could be explained by common pig breeding lines and by the intense commercial trade of pigs and products thereof between countries [33]. Additionally, a third less frequent MDR clone of *S.* 1,4,[5],12:i:-, Southern-European clone/ST19, was reported in Portugal [24,82] and sporadically in Italy and Spain [83]. 

*S.* Derby and *S.* Rissen have been other predominant serotypes in both pig and pork meat in Europe [6,7,8], and in USA [84], despite being less implicated in human salmonellosis. Nevertheless, *S.* Derby has been reported at global level with identical MDR (mainly SSuT) and/or PFGE profiles in isolates from human clinical cases, pigs and products thereof, demonstrating their potential role in human infections [19,20,52,62,63,65,85,86,87,88]. In Southern European countries, *S.* Rissen is considered a clinically-relevant serotype, being frequently detected the same strains in humans, pigs and products thereof [19,21,22,25,27,62,65,89,90]. Moreover, *S.* Rissen strains detected among humans and across pig production chain, particularly belonging to the successful MDR clone ST469, have been reported in geographic distant countries [16,19,21,22,27,62,63,65,89,90]. In particular, the circulation of a specific MDR (ASSuTTm, Trimethoprim-Tm) *S.* Rissen clone between the Iberian countries can be explained by the intensive trade of pigs and products thereof [21]. Additionally, some *S.* Rissen isolates recovered from human, pig and pork isolates in Denmark showed similar PFGE profiles with isolates from imported pigs or pork meat from Spain and Germany, as well as with isolates from human clinical cases of people who travelled to Thailand [27] (Table 1). These data enhance the contribution of live animals and international food trade to the spread of this *S.* Rissen clone, besides human travel to developing countries. 

The enhanced ability to colonize food animals and to persist along the food chain of pig-related *Salmonella* serotypes and clones associated with human infections is a topic of great concern [26,64,65]. Specific adaptive features, such as colonization/virulence determinants, have been pointed out as an advantage for the maintenance and spread of these serotypes/clones in diverse environments and hosts (pig/human) [116]. Recent studies have found the presence of several virulence genes, associated with an enhanced adaptation to the food-animal host, in *S.* Typhimurium [116,117], in specific clones of *S.* 1,4,[5],12:i:- strains circulating in Europe [72,73,80,118] and in *S.* Rissen, including in isolates belonging to the ST469 [119,120]. Those virulence genes encode for proteins that improve colonization (e.g., *clpB*), adhesion (e.g., *csgA*, *fimA/C*, *pefA*, *stbD, marT*), intestinal invasion (e.g., *invA*, *invE*, *spvC*), survival in host tissues (e.g., *sopA*, *avrA*, *sseI*, *mig5*) and biofilm formation (e.g., *bss*) [83,117,119,121,122]. Interestingly, *S.* Typhimurium DT193 and *S.* 1,4,[5],12:i:- were associated with long-term survival in pig faeces comparing with other serotypes (*S.* Derby and *S.* Bredeney), due to their increased adaptation to acid fecal pH and organic acid supplementation of feed [123]. Additionally, a UK study demonstrated that SPI-23 present in *S.* Derby strains, which contain genes (e.g., *potR*) that encode Type III effector proteins, contributes for the host intestinal cells invasion in pigs [124]. More recently, the presence of this SPI-23 was also reported in French pork isolates of *S.* Derby ST39 and ST40, helping to explain the host pig specificity of those epidemic strains [125].

Moreover, those emergent pig-related *Salmonella* serotypes/clones were usually enriched with antimicrobial resistance genes, in most cases located in the mobile genetic elements that also carry virulence genes. For example, resistance plasmids of *S.* 1,4,[5],12:i:- isolates (from pigs and humans) circulating in Europe, carry several virulence genes, namely *spvC*±*mig5* genes in IncA/C and IncR plasmids, associated with the Spanish and Southern European clones, respectively [83]. Furthermore, several genes associated with tolerance to metals and/or biocides (e.g., copper), widely used in food-animal production, were found in pig-related *Salmonella* serotypes/clones (e.g., *S.* Rissen MDR clone, European clone of *S.* 1,4,[5],12:i:- and *S.* Typhimurium), which might also be an additional advantage for their maintenance and spread in the food production environment and hosts (pig/human) [25,126,127].

## 5. Antimicrobial Resistance in *Salmonella* and the Pork Linkage

Antibiotic resistance is considered by several relevant public health entities one of the major threats to human health and a relevant concern for food safety, particularly if involves pathogenic bacteria transmitted to humans through food-chain [3,26]. The emergence and spread of *Salmonella* isolates presenting resistance to several antibiotics, especially to “Highest Priority Critically Important Antimicrobials” (fluoroquinolones and 3rd and higher generations cephalosporins) [3], is of concern since they are crucial to the successful treatment of NTS invasive infections [1,2]. The adverse consequences of resistance to critically important antibiotics in humans include an increase in the severity of infections and in the frequency of treatment failures, as well as the requirement of last-line antibiotics use (e.g., carbapenems, colistin) [3,26].

The common practice of antibiotic use in intensive food-animal production has been considered the main driver for the selection and transmission of antibiotic-resistant foodborne bacteria, including *Salmonella,* to humans [1,26,128,129,130,131,132]. This scenario is aggravated particularly in pig production, which has been associated with a higher antimicrobial consumption, compared with other animal-food production systems, at global level [133], including in the EU [134]. In 2010, the annual average of antimicrobial consumption per kilogram of animal produced was 172 mg·kg^−1^ in pigs, higher than the 148 mg·kg^−1^ and 45 mg·kg^−1^ consumption in chicken and cattle, respectively [133]. Although there is still controversy about the contribution of food-animal reservoirs and food vehicles in the transmission of antibiotic-resistant bacteria with an impact in human health, there is accumulating evidence linking the pig production with antimicrobial resistance in NTS that will be discussed in the next sections. 

### 5.1. Association between Antibiotic Use in Pig Production and Resistance in Salmonella

The first evidence of this linkage is the association between the amount and pattern of antimicrobial agents used in the pig production and the occurrence of resistant NTS in pigs, pork meat and/or humans. One illustrative case in pig production includes a study showing that the administration of tetracycline to pigs colonized with tetracycline-resistant *S.* Typhimurium DT104 was associated with higher pig shedding of this resistant strain compared with untreated pigs [135]. Other study reported that enrofloxacin, used for treatment of pigs, induced the selection of *S.* Typhimurium with decreased susceptibility to ciprofloxacin [136]. Furthermore, a Danish surveillance study performed after the ban of antibiotics as growth promoters showed higher levels of tetracycline resistance in *S.* Typhimurium isolates recovered from pig and human clinical cases, potentially caused by an increased usage of tetracycline in pigs [137].

### 5.2. Correlation of Antimicrobial Resistance Rates between Salmonella from Pigs and Humans

The correlation between antibiotic resistance rates among *Salmonella* from pigs, pork meat and humans obtained from systematic surveillance data evidences the impact of pig production practices on NTS antibiotic resistance. The 2016 EFSA report showed a high prevalence of MDR *Salmonella* in humans (29.3%), pigs (58.7%) and pork meat (40.4%), including the most used antibiotics in pig production (tetracyclines, penicillin’s, sulphonamides and colistin) [26,134]. In fact, high levels of resistance to A-27.8%, Su-32.4% and T-28.1% and MDR-29.3% were observed in *Salmonella* from human isolates, as well as from pigs (A-45.3%; Su-52.6%; T-53.5%; MDR-43.9%) and pork meat (A-44.7%; Su-48.5%; T-49.1%; MDR-40.4%). Furthermore, the ASuT phenotype was the most frequent MDR profile observed in pig and pork meat (82.3% and 80%, respectively), with the majority of the isolates belonging to the pig-related serotype *S.* 1,4,[5],12:i:- (66.4%-pig and 69.6%-pork meat) [26]. Indeed, high levels of MDR were observed in pig-related serotypes, namely *S.* 1,4,[5],12:i:- (MDR = 81.1%-humans; 82.3%-pigs; 73.8%-pork meat), *S.* Typhimurium (44.4%-humans; 52.4%-pigs; 54.5%-pork meat), *S.* Derby (23.8%-humans; 20.3%-pigs; 10.4%-pork meat) and *S.* Rissen (33.3%-pigs; 52.8%-pork meat). Additionally, this report highlights that those pig-related serotypes were the major contributors to the observed prevalence of resistance in *Salmonella* in both pig and pork meat samples. From 2014 to 2015 MDR in humans increased more than 10% in both *S.* Typhimurium and *S.* 1,4,[5],12:i:- serotypes [26]. In the USA, data from the National Antimicrobial Resistance Monitoring System (NARMS) showed an increase of the ASSuT phenotype in *S.* 1,4,[5],12:i:- from human (from 43% in 2014 to 60% in 2015), and swine isolates (65% in 2015) [138]. 

Data about MDR phenotypes are of concern due to the possible role of diverse antibiotics in the co-selection of *Salmonella* strains resistant to clinically-relevant ones, such as fluoroquinolones (e.g., ciprofloxacin-Cp), extended-spectrum cephalosporins (e.g., ceftazidime-Caz, cefotaxime-Ctx) and colistin (Col) [3,26,131,134]. In the last EU report, relatively low levels of *Salmonella* resistance to Cp (13,3%), Caz (0.9%), Ctx (0.9%), and Col (11.4%) were observed [26,139]. Moreover, the highest levels of Col resistance in humans (excluding the *S.* Enteritidis serotype which presents intrinsic resistance) were more common in the pig-related serotypes *S.* 1,4,[5],12:i:- (2.4%) and *S.* Typhimurium (1.5%) [26,139]. Additionally, data from 2015 in USA revealed low levels of decreased susceptibility to Cp (5.8%) among humans, being the highest levels detected in retail pork samples (5.3%) comparing with other retail meat (0% in poultry and beef) [140], which suggest an important contribution of pig production chain to the human disease burden.

In contrast, high rates of antibiotic resistance were reported among humans and pig/pork products in middle-income countries. For instance, high rates of *Salmonella* with resistance to Cp (15–48%), ceftriaxone-Cro (38%) and Col (36%) were observed in humans [141,142,143]. High levels of resistance to clinically-relevant antibiotics were also observed in pigs or pork meat, in particular resistance to Cp (10–49.4%) [144,145,146] and Col (7–21%) [142,147], being in most cases higher than those detected in poultry samples (Cp-0–29%, Col-3.8%) [142,144,146]. Moreover, different Chinese studies reported higher rates of acquired Col resistance mechanisms (Mobile Colistin Resistance—MCR) in pigs (10–14.8%) than in poultry samples (0.8–7.5%) [148,149,150]. Indeed, it was already suggested that pig production has the highest impact on colistin resistance in humans because of its extensive use in this system contrasting with other food-producing animals [151,152]. Overall, the high incidence of resistance to clinically-relevant antibiotics observed in *Salmonella* from pig production highlights the potential role of pork products in its spread and may be the consequence of the high, inappropriate or uncontrolled use of antibiotics in farming practices in middle-income countries [133,143,147,150,153,154]. In fact, it was estimated that the global consumption of antimicrobials in livestock will increase by 67% between 2010 and 2030, with an expected increase in pigs of 124%, particularly in Asia, in order to respond to the increasing demand of pork meat, one of the most consumed and traded meat products [30,133,155]. Hence, international trade of live pigs (piglets, weaner, grower or breeder pigs) and pork meat can contribute for the worldwide spread of antibiotic-resistant *Salmonella,* with a consequent impact on human health. 

### 5.3. Transmission of Antimicrobial Resistant Salmonella from Pork Meat to Humans

The presence of the same mobile genetic elements carrying clinically-relevant antibiotic resistance genes and/or antibiotic-resistant *Salmonella* serotypes/clones in pig and human isolates is an additional evidence of transmission of antimicrobial resistant *Salmonella* from pork meat to humans. In the last decades, clinically-relevant antibiotic resistance genes such as those coding for extended-spectrum β-lactamases (ESBLs), acquired AmpC β-lactamases (qAmpC), plasmid-mediated quinolone resistance (PMQR) and more recently plasmid-mediated colistin resistance (MCR), have been globally reported in a wide range of *Salmonella* serotypes associated with pig and pork products (Table 2). Additionally, some examples that illustrate the linkage and transmission of MDR *Salmonella* from pigs/pork products to humans are shown in Table 2. For instance, as examples of strains/clones shared between pork and human were *S.* Typhimurium with *bla*_CTX-M-1_, *bla*_CMY-2_ or *oqxAB*±*aac(6′)-Ib-cr*, *Salmonella* Virchow with *bla*_CTX-M-15_, and *S.* Typhimurium, *S.* 1,4,[5],12:i:- or *S.* Bovismorbificans carrying *mcr-1* gene (Table 2). 

The most prevalent genes coding for resistance to extended-spectrum cephalosporins were those coding for CTX-M enzymes (e.g., CTX-M-1, CTX-M-14, CTX-M-15) followed by qAmpC CMY-2, both reported in pigs/pork products and humans, and increasingly associated with pig-related serotypes (e.g., *S.* Typhimurium and *S.* 1,4,[5],12:i:-) (Table 2) [131,156]. Plasmid-mediated quinolone resistance (PMQR) mechanisms have been also widely reported in diverse NTS serotypes from both pig/pork products and human isolates [156,157,158]. The most frequent PMQR were QNR proteins (e.g., QnrB19, QnrS1/S2) found in diverse regions and serotypes (Table 2). Also the aminoglycoside acetyltransferase AAC(6′)-Ib-cr, commonly associated with the efflux pump OqxAB, was widely reported among relevant pig-related NTS serotypes (*S.* Typhimurium, *S.* Derby and *S.* Rissen), particularly in Asian countries (Table 2). Finally, pig/pork seems to be an important vehicle of NTS carrying the emergent plasmid-mediated colistin resistance mechanism, (mostly MCR-1) (Table 2), also predominantly in pig-related NTS serotypes causing human infections [75,153,156,159].

All these data demonstrate that pig/pork products might be an important vehicle for the transmission and dissemination of NTS carrying acquired clinically-relevant resistance genes to humans through the food chain. The most frequent and clinically-relevant antibiotic resistance genes and their associated genetic elements (such as specific plasmids) reported in pigs/pork products were also found in NTS human isolates (Table 2). In fact, transmission of the most commonly reported genes in both sources has been associated with several epidemic plasmid families, such as IncN (*bla*_CTX-M-1_, _-27_, _-65_), IncFIB (*bla*_CTX-M-14_, _-27_, _-65_, *bla*_CMY-2_), IncA/C (*bla*_CTX-M-27_, *bla*_CMY-2_), IncI1 (*bla*_CTX-M-1_, *bla*_CMY-2_), IncHI2 (*bla*_CTX-M-1_, _-14_, _-15_, *oqxAB*±*aac(6′)-Ib-cr*, *mcr-1*), IncI2 or IncX4 (*mcr-1*) (Table 2) [131,153,156,157]. More worrying is the presence, in the same *Salmonella* strains, of different clinically-relevant antibiotic resistance genes (e.g., *mcr-1*+*bla*_CTX-M-1_, *mcr-1*+*oqxAB+aac(6′)-Ib-cr*) co-located in the same genetic elements [150,160] (Table 2), worsening the possibility of clinical treatment failure of invasive NTS infections. 

The acquisition of resistance mechanisms to antibiotics commonly used in food-animal production (e.g., ampicillin, sulphonamides, tetracyclines) is also relevant for their potential role in the co-selection of pig-related MDR *Salmonella* clones in pig production and further transmission to humans. Several studies have provided evidence of successful transmission of MDR *Salmonella* clones from diverse serotypes from pork to humans [19,20,23,24,161,162]. For example, we recently reported *mcr-1* located on epidemic plasmids (IncX4 and IncHI2) in clinically-relevant MDR *S.* 1,4,[5],12:i:-/ST34 [159]. Additionally, in China, diverse studies reported the clonal spread of *mcr-1*+*oqxAB*+*aac(6′)-Ib-cr* in *S.* Typhimurium/ST34 in pigs presenting resistance to multiple antibiotics (e.g., A, S, Su, T) [149,150]. Moreover, some of these successful clones presented acquired metal tolerance genes, an additional feature that might be contributing for the survival and persistence of these strains in metal contaminated environments, such as the pig production setting [25,126,127,163]. For example, *sil±pco* genes encoding for copper/silver tolerance were often found in the emergent European clone of *S.* 1,4,[5],12:i:- and *S.* Typhimurium, as well as *S.* Rissen/ST469 MDR clone [25,126,127]. In fact, copper is one of the most used metal compounds in pig setting (e.g., as supplements in animal feed), suggesting that in these environments higher selective pressures could contribute for the co-selection of MDR NTS clones [25,126,127,159,163,164,165], with consequences for food safety and human health.

The data presented here show that pig production setting can be a relevant reservoir of successful and worldwide emergent MDR pig-related *Salmonella* serotypes/clones, enriched with different adaptive features (e.g., metal/biocides tolerance genes) besides genes conferring resistance to critical antibiotics, which might spread to humans through the food chain. Moreover, the increasing trend of intensive pig production systems and the globalization of pork products pose a major challenge for the spread of antimicrobial resistance in zoonotic bacteria such as NTS, with a consequent impact on human health. Therefore, it is crucial to restrain the use of antimicrobial agents in pig production and to improve biosecurity measures (e.g., high standards of hygiene, regular veterinary checks, vaccination), aiming to minimize selection and spread of MDR clones along all stages of pig production chain. 

## 6. Conclusions

Pork products are among the most frequent foodstuffs implicated in human salmonellosis, with pig and pork meat reported worldwide as important sources of NTS resistant to clinically-relevant antibiotics, representing a major threat to the treatment of invasive infections. Furthermore, the high incidence of resistance to clinically-relevant antibiotics reported in diverse countries together with the increasing demand for pork meat and the global trade of pig/pork products raised the current public health concern. Hence, the continuous control and monitoring of *Salmonella*, particularly targeting specific MDR pig-related serotypes/clones, along the food chain (from primary production to consumption) is critical to minimize their introduction in the food-animal production and further transmission to humans. Therefore, a global integrated surveillance (“One Health” approach), and the implementation of more effective measures are critically needed, including the improvement of biosecurity measures at farms (e.g., providing uncontaminated feed, isolation of new purchased animals, high standards of hygiene, regular veterinary checks, vaccination), during slaughtering/processing (e.g., prevent external sources of contamination during transport, lairage and slaughter, cross-contamination with equipment and workers and hygiene practices such as cleaning and disinfection) and retail/consumer level (e.g., avoiding cross-contamination, using safe cooking temperature). Surveillance of antibiotic resistance levels in NTS throughout the pig food chain is crucial to ensure public health, not only through the detection of new food safety risks involving foodstuffs such as pork meat but also to avoid salmonellosis treatment failures.

## Figures and Tables

**Table 1:** *Salmonella* outbreaks linked to pork meat products (2004–2018).

Serotype	Country(ies) ^2^	Source	Year(s)	No. of ^3^		Reference
(Subtyping features/molecular markers, when available) ^1^				**Cases**	**Death(s)**	
**Typhimurium**						
(DT104A)	Italy	Pork salami	2004	63	0	[91]
(DT12)	Denmark	Pork products	2005	26	0	[92]
(MLVA-type 3-12-4-13-2)	Denmark, Norway, Sweden	Danish pork meat/minced meat	2008	37/10/4	4/0/0	[93]
(DT193; MLVA-type 3-14-12-NA-211)	Denmark	Pork salami	2010	20	NS	[94]
(DT120; MLVA-type 3-11-14-NA-211)	Denmark	Imported smoked pork tenderloin	2011	22	0	[95]
(DT193)	Spain	Dried pork sausage	2011	8	0	[96]
	Australia	Barbecued pork	2010	4	NS	[97]
	Australia	Pork spit roast	2011	5	NS	[97]
	Australia	Cooked pork hock	2014	4	NS	[97]
	Australia	Pork dish	2015	10	NS	[97]
(ST19)	Denmark ^4^	Spanish salami	2017	NS	NS	[98]
	Sweden	Spanish salami	2017	NS	NS	[98]
**1** **,4,[5],12:i:-**						
(DT193; PFGE-type STYMXB.0131; ASSuT)	Luxemburg	Pork meat	2006	133	1	[99]
(MLVA-Type 3-13-15-NA-211)	France	Dried pork sausage	2010	69	0	[100]
(PFGE-type XTYM-159; MLVA-type 3-13-9-NA-211; ASSuT)	France	Dried pork sausage	2011	337	0	[101]
(PFGE-type STYMXB.0131/ STYMXB.0083; MLVA-type 3-13-9-NA-211; ASSuT)	Italy	Pork salami	2012–2015	NS	NS	[102]
(DT138; PFGE-type XbaI.0005/ STYMXB.0083; BlnI.00X; ASSuT)	Spain	Pork chorizo	2014	6	0	[103]
(DT138)	Spain	Dried pork sausage	2011	38	0	[96]
	USA	Pork meat	2015	188	0	[60]
	Sweden ^4^	Italian chilled truffle salami	2018	NS	NS	[98]
**Derby**						
	Spain	Dried pork sausage	2011	3	0	[96]
(PT53; ST682)	Germany	Raw fermented pork	2013–2014	145	0	[104]
**Rissen**						
(PFGE-type TEEX01.0017.DK)	Denmark ^4^	Imported pork products	2000–2005	NS	NS	[27]
**Bovismorbificans**						
(PT24)	Germany	Pork minced meat	2004–2005	525	1	[105]
(ST142)	The Netherlands, Belgium, France	Pork ham products	2016-2017	54/NS/NS	0/NS/NS	[106]
**Give**	Germany	Minced pork meat	2004	115	1	[107]
**Goldcoast**						
(PFGE-type SCG Xba^3^)	Hungary	Pork	2009–2010	44	0	[108]
	Italy	Pork salami	2009–2010	79	0	[109]
**Infantis**						
(PFGE-type EPI-type)	Denmark	Pork meat	1992–1993	>500	0	[110]
(ST32)	Italy	Pork meat—porchetta	2011	23	0	[111]
(PT29; PFGE-type XB27)	Germany	Raw pork	2013	267	0	[112]
	Australia	Pork rools	2013	2	NS	[61]
	USA	Pork meat	2015	5	0	[60]
**Manhattan**	France	Pork products	2005–2006	69	0	[113]
**Muenchen**						
	Germany	Raw pork	2013	203	0	[114]
	Germany	Raw pork	2014	247	0	[114]
**Ohio**						
(PFGE-type BlnI-A)	Belgium	Pork meat	2005	6o	0	[115]

^1^ Antimicrobial compounds: A, ampicillin; S, streptomycin; Su, sulphonamide compounds; T, tetracycline. DT/PT, Phage type; MLVA, Multiple Locus Variable-number Tandem Repeat Analysis; NA, designates a locus that is not present; PFGE, Pulsed-field Gel Electrophoresis; ST, Sequence type. ^2^ Only outbreaks in USA, EU and Australia are shown. ^3^ Estimated number of cases only when outbreaks were reported. ^4^ Suspected outbreaks.

**Table 2:** *Salmonella* serotypes/clones carrying clinically-relevant antibiotic resistance genes recovered from pig and products thereof.

Serotype	Clinically Relevant Gene(s) ^1^ (no. isolates)	Source	Human Concomitant Presence ^2^	Country/year(s)	Antibiotic Resistance Phenotype ^3^	Gene Location (PL-Inc Group/Chr)	Reference
**Typhimurium**	*bla*_TEM-52_ (n = 2)	Pigs		Belgium/2009	NR	NR	[166]
	*bla*_CTX-M-1_ (n = 1)	Pork meat		Germany/2007	K-N-S-Su-T-Tm-Sxt	PL-N	[167]
	*bla*_CTX-M-14_+*bla*_SHV-1_+*oqxAB*+ *aac(6′)-Ib-cr* (n = 2)	Pigs	Yes	China/2014	NS	PL-FIB, N	[168]
	*bla*_CTX-M-27_+*bla*_SHV-1_+ *oqxAB*+ *aac(6′)-Ib-cr* (n = 1)	Pig		China/2014	NS	PL-P	[168]
	*bla*_CTX-M-27_+*qnrB*+*oqxAB*+ *aac(6′)-Ib-cr* (n = 1)	Pig		China/2014	NS	PL-NT	[168]
	*bla*_CTX-M-55_+*qnrA*+*qnrB* (n = 4)	Pigs		China/2016	A-(Cp-E-F-L-Na-N-T)	NR	[58]
	*bla*_CTX-M-65_+*oqxAB* (n = 1)	Pig		China/2014	NS	PL-FIB	[168]
	*bla*_CMY-2_ (n = 52)	Pork		Colombia/UN	A-T	NR	[169]
		Pork and pig	Yes	Mexico/2002–2005	C-S-Su-T-(G-K-Na-Sxt)	NR	[170]
		Pigs		Mexico/NR	A-Caz-Ctx-Fox-T-(G)	NR	[46]
		Pigs		USA/2007	A-C-Cro-Fox-G-K-S-Su-Sxt-T-Ti	PL-A/C, FIB, I1	[171]
		Pigs		Belgium/2009	NR	NR	[166]
		Diarrheic pigs		South Korea/2011–2012	A-Cef-C-F-Fox-G-Na-Sxt-T-Ti	PL-A/C, FIB	[172]
	*qnrS1*+*oqxAB*+*aac(6′)-Ib-cr* (n = 4)	Pork		China/2012–2013	A-C-Cp-G-K-Na-O-S-Su-T	NR	[173]
	*oqxAB* (n = 3)	Pigs		China/2010	A-C-F-Na-O-Su-T-(G)	PL-F	[174]
		Pork		China/2012–2013	A-C-Cp-K-Na-O-S-Su-T-(G)	NR	[173]
	*oqxAB*+*aac(6′)-Ib-cr* (n = 6)	Pigs		China/2008–2010	A-Na-O-Sxt-T-(C-Cp-F-G)	PL-HI2	[174]
		Pork		China/2012	A-C-Cp-G-K-Na-O-S-Su-T	NR	[173]
	*mcr-1* (n = 13)	Pigs		Spain/2009–2011	Col	PL-NR	[175]
		Ready-to-eat pork		China/2014	C-Cp-Col-F-G-Na-S-Sxt-T	PL-HI2	[176]
		Pork carcass/ Pork product		Portugal/2016	A-Col-S-Su-T-Tp-(C-Cp-Fox)	PL-HI2	[177]
		Pig	Yes	Great Britain/NR	A-C-Col-Fox-Su-T-Tm-Tyg	PL-I2	[178]
	*mcr-1*+*bla*_CTX-M-1_ (n = 1)	Pig		Portugal/2011	A-At-C-Caz-Col-Ctx-Fep-Fox-G-T-Tob	PL-HI2	[160]
	*mcr-1*+*bla*_CTX-M-14_ (n = 2)	Pork		China/2015	A-C-Caz- Col-Ctx-Fos-G-Su-T	PL-HI2	[179]
	*mcr-1*+*oqxAB* (n = 17)	Pigs		China/2013–2014	Cp-Col-F-O-Sxt-T-(A-G-S)	PL-HI2-F4:A-:B5 ^4^	[150]
	*mcr-1*+*oqxAB*+*aac(6′)-Ib-cr* (n = 4)	Pigs		China/2008–2009	A-Col-F-G-Na-O-S-Sxt-T-(Cp)	PL-I2, HI2	[149]
	*mcr-1*+*aac(6′)-Ib-cr* (n = 1)	Pig		China/2008–2009	A-Col-F-G-Na-O-S-Sxt-T	PL-I2	[149]
		Pigs		China/2015	A-Cp-Col-G-(C-Na-Sxt)	PL-HI2	[179]
**1** **,4,[5],12:i:-**	*bla*_CTX-M-1_ (n = 15)	Pigs		UK/2009	A-Ctx-Su-(C)	PL-I1-γ	[180]
		Pigs		Germany/2007, 2009–2010	A-At-Cro-Ctx-Cef-Cxm-P-Tc-Ti-(Fep-S)	PL-N, I1	[181]
	*bla*_CTX-M-14_ (n = 1)	Pork		Portugal/2010	A-C-Ctx-T-Tm	NR	[182]
	*bla*_CTX-M-15_+*bla*_SHV-12_ (n = 1)	Pork		Portugal/2011	A-C-Ctx-S-Su	NR	[182]
	*bla*_CTX-M-32_ (n = 1)	Pork		Portugal/2011	A-C-Ctx-G-S-Su-T	NR	[182]
	*qnrB19* (n = 1)	Pig		USA/2014	Cp	PL-NR	[183]
	*mcr-1* (n = 24)	Pigs	Yes	Italy/2012–2015	A-Col-S-T-(C-Cp-F)	NR	[75]
		Pork	Yes	Italy/2013–2015	A-Col-S-T-(C-F)	NR	[75]
		Pork carcass/ Pork meat	Yes	Portugal/2014–2015	A-Col-S-Su-T-(C-Cp-Tm)	PL-X4, HI2	[159]
		Slaughterhouse/ Pork sausage		Portugal/2015–2016	A-Col-S-Su-T-(Cp-Tm)	PL-X4, HI2	[177]
		Pork carcass		France/2016	Col	PL-NR	[184]
		Pork carcass		Belgium/2012	A-Col-S-Su-T	PL-X4	[185]
	*mcr-4* (n = 1)	Pig		Italy/2014	A-Col-S-Su-T	PL-ColE	[186]
**Derby**	*bla*_CTX-M-1_ (n = 3)	Pigs		Belgium/2009	NR	NR	[166]
		Pork sector		France/2014	A-Cef-Ctx-Caz	NR	[125]
	*qnrA* (n = 2)	Pigs		China/2016	A	NR	[58]
	*qnrB19* (n = 3)	Pigs		USA/2014	Cp	PL-NR	[183]
		Pork chops		USA/2014–2015	Cp	PL-NR	[183]
	*qnrB*+*qnrS1*+*oqxAB* (n = 1)	Pork		China/2013	A-C-Cp-G-K-Na-O-S-Su-T-(Az)	NR	[173]
	*qnB8*+*qnrS2*+*oqxAB*+ *aac(6′)-Ib-cr* (n = 1)	Pork		China/2012	A-C-Cp-G-K-Na-O-S-Su-T	NR	[173]
	*qnrS2* (n = 1)	Pork chops		USA/2014	Cp	NR	[183]
	*qnrS2*+*oqxAB*+*aac(6′)-Ib-cr* (n = 11)	Pork		China/2012–2013	A-C-Cp-G-K-Na-O-S-Su-T-(Az)	NR	[173]
	*oqxAB* (n = 3)	Pork		China/NR	C-Na-O-T	Chr	[187]
		Pork		China/2013	A-C-Cp-G-K-Na-OLA-S-Su-T	NR	[173]
	*mcr-1* (n = 13)	Pigs		Italy/2012–2015	C-Col-S-Su-Sxt-T	NR	[75]
		Pork sausage		France/2013	Col-(A-S-T)	PL-P	[188]
		Pork		China/2015	A-C-Col-T	PL-X4	[179]
	*mcr-2* (n = 1)	Pork carcass		Belgium/2012	C-Col-Su-Tm	PL-X4	[185]
**Rissen**	*bla*_CTX-M-1_ (n = 1)	Pig		Belgium/2009	NR	NR	[166]
	*bla*_CTX-M-55_ (n = 1)	Pork carcass		Thailand/2014–2015	A-C-Caz-Ctx-Cpd-G-S-Su-T	PL-NR	[50]
	*bla*_SHV-12_ (n = 1)	Pig		Spain/1999	A-At-Caz-Cef-Ctx-S-Su-T	NR	[189]
	*qnrB19* (n = 1)	Pig		USA/2013	Cp	PL-NR	[183]
	*qnrS1* (n = 1)	Pig		Korea/2012–2013	Cp-Na	NR	[57]
	*qnrVC4* (n = 1)	Pig		Thailand/2007	A-C-Cp-Na-S	PL-Q1	[190]
	*oqxAB* (n = 1)	Pork		China/2013	A-C-Cp-K-Na-O-S-Su-T	NR	[173]
	*mcr-1* (n = 3)	Pig		Spain/2009–2011	Col	PL-NR	[175]
		Pork carcass		Portugal/2014–2015	A-C-Col-S-Su-Tm-(T)	PL-X4	[159]
**Anatum**	*bla*_CMY-2_ (n = 4)	Pigs		USA/2008-2011	A-Cro-Fox-Su-T-Ti	PL-I1-γ	[191]
	*qnrB19* (n = 5)	Pigs		USA/2014	Cp	PL-NR	[183]
	*mcr-1*+*bla*_CMY-2_ (n = 3)	Pigs		Taiwan/2013	A-C-Caz-Cp-Col-Ctx-Fox-Na-S-Su-Sxt-T	PL-NR	[142]
**Adelaide**	*qnrB19* (n = 1)	Pig		USA/2014	Cp	PL-NR	[183]
**Bovismorbificans**	*bla*_CTX-M-1_ (n = 1)	Pig		UK/2009	A-Ctx-Su	PL-I1-γ	[180]
	*mcr-1* (n = 1)	Pork	Yes	Italy/2013-2015	A-C-Cp-Col-S-T	NR	[75]
**Brandenburg**	*qnrB19* (n = 1)	Pigs		USA/2014	Cp	PL-NR	[183]
**Concord**	*qnrB2* (n = 1)	Pig		Czech Republic/NR	NR	NR	[158]
**Dublin**	*mcr-1-like* (n = 1)	Pig		France/NS	NS	NR	[192]
**Enteritidis**	*bla*_CTX-M-15_ (n = 2)	Pigs		Korea/2012-2013	A-Cep-G-Na-N-S-T-Ti	PL-HI2	[57]
	*bla*_CTX-M-55_+*qnrA+qnrB* (n = 2)	Pigs		China/2016	A-(Cp-F-Fos-Na-T-Ti)	NR	[58]
	*qnrA+qnrB* (n = 1)	Pig		China/2016	A-Cp-F-Na	NR	[58]
	*qnrS1/S3* (n = 1)	Pig		Poland/2008	A-Cp	NR	[193]
**Give**	*bla*_CMY-2_ (n = 1)	Pig		USA/1998-1999	A-At-C-Caz-Ctx-Fox-G-P-S-Su-T-Tc-Ti	PL-NR	[194]
**Goldcoast**	*qnrS1* (n = 1)	Pig		Belgium/NR	NR	NR	[158]
**Heidelberg**	*bla*_CMY-2_ (n = 3)	Pigs		Canada/2004	A-Cef-Fox-Ti	PL-NR	[195]
		Pigs		USA/1998-1999	A-At-C-Caz-Ctx-Fox-G-P-S-Su-Sxt-T-Tc-Ti	PL-NR	[194]
	*mcr-1*+*oqxAB* (n = 1)	Pig		China/2013-2014	Cp-Col-F-O-S-Sxt	PL-HI2-F4:A-:B5 ^4^	[150]
**Hinsingen**	*qnrD*+*aac(6′)-Ib-cr* (n = 1)	Pig		China/2009-2010	Cp-Na	PL-NR	[196]
**Indiana**	*bla*_CTX-M-27_+*bla*_SHV-1_+*oqxAB* (n = 2)	Pigs		China/2014	NS	PL-N, P	[168]
	*bla*_CTX-M-65_+*bla*_SHV-1_+*oqxAB*+ *aac(6′)-Ib-cr* (n = 1)	Pig		China/2014	NS	PL-NT	[168]
	*bla*_CTX-M-65_+*oqxAB*+ *aac(6′)-Ib-cr* (n = 7)	Pig		China/2011	A-C-Caz-Cp-Ctx-G-Na-Sxt-T	PL-NR	[144]
		Pork		China/2012	A-Az-C-Cp-Cro-G-K-Na-O-S-Su-T	NR	[173]
		Pig		China/2014	NS	PL-N	[168]
	*qnrA*+*oqxA*+*aac(6′)-Ib-cr* (n = 1)	Pig		China/2009-2010	Cp-O-Na	PL-NR	[196]
	*oqxA* (n = 2)	Pig		China/2009-2010	Cp-O-Na	PL-NR	[196]
	*aac(6′)-Ib-cr* (n = 1)	Pig		China/2009-2010	Cp-Na	PL-NR	[196]
**Infantis**	*bla*_CMY-2_ (n = 2)	Pigs		Japan/2007-2008	A-C-Caz-Cef- Fox-S-Su-T	PL-NR	[59]
	*bla*_VIM-1_+*bla*_AAC-1_ (n = 4)	Pigs		Germany/2011	A-C-Caz-Cef-CRro-Ctx-Cxm-Fep-Fox-P-S-Su-Tc-Ti-Tm	PL-HI2	[197]
**Kedougou**	*mcr-4.1* (n = 1)	Pig carcass		Spain/2016	Col	PL-NR	[184]
**London**	*bla*_CTX-M-14_ (n = 1)	Pork		Portugal/2012-2013	A-Ctx	NR	[198]
	*qnrB19* (n = 1)	Pig		USA/2014	Cp	PL-NR	[183]
	*mcr-1*+*oqxAB* (n = 1)	Pig		China/2013-2014	A-Cp-Col-F-G-O-S-Sxt	PL-HI2-F4:A-:B5 ^4^	[150]
**Miami**	*bla*_CMY-2_ (n = 2)	Diarrheic piglets		India/2014	A-Cfl-Ctx-E-Fix-P	NR	[199]
**Muenchen**	*qnrB19* (n = 8)	Pigs		USA/2013-2014	Cp	PL-NR	[183]
**Newport**	*mcr-1*+*bla*_TEM-135_ (n = 5)	Pigs		China/2015	A-C-Cp- Col-G	PL-HI2	[179]
**Senftenberg**	*qnrB6*+*aac(6′)-Ib-cr* (n = 1)	Pig		USA/2013	Cp	NR	[183]
**Virchow**	*bla*_CTX-M-15_ (n = 5)	Pigs	Yes	Korea/2012-2013	A-Cef-G-Na-N-S-T-Ti	PL-HI2	[57]
**Weltevreden**	*mcr-1* (n = 1)	Pork		China/2015	Col-T	PL-X4	[179]
***Salmonella*** **spp.**	*mcr-1* (n = 3)	Pigs		Italy/2010-2011	NR	NR	[192]

Antimicrobial abbreviations: A, ampicillin; At, aztreonam; Az, azithromycin; C, chloramphenicol; Caz, ceftazidime; Cpd, cefpodoxime: Cef, cephalotin, Cfl, cephalexin; Cp, ciprofloxacin; Col, colistin; Cro, ceftriaxone; Ctx, cefotaxime; Cxm, cefuroxime; E, Enrofloxacin; F, florfenicol; Fep, cefepime; Fix, cefixime; Fos, fosfomycin; Fox, cefoxitin; G, gentamicin; K, kanamycin; L, levofloxacin; N, neomycin; Na, nalidixic acid; O, olaquindox; P, piperacillin; S, streptomycin; Su, sulphonamide compounds; Sxt, trimethoprim/sulfamethoxazole; T, tetracycline; Tc, ticarcillin; Ti, ceftiofur; Tm, trimethoprim; Tob, tobramycin; Tyg, tigecycline. Chr, chromosome; NS, not specified; NR, not reported; NT, not typable; PL, plasmid. ^1^ Only references with full-characterized clinically relevant antibiotic resistance genes were considered. ^2^ “Yes”, clones or serotypes detected both in pigs/products thereof and humans in the same study. ^3^ Variable phenotypes were present between curved brackets. ^4^ IncF replicon sequence typing.
